# Audio deepfakes: A survey

**DOI:** 10.3389/fdata.2022.1001063

**Published:** 2023-01-09

**Authors:** Zahra Khanjani, Gabrielle Watson, Vandana P. Janeja

**Affiliations:** Department of Information System, University of Maryland Baltimore County, Baltimore, MD, United States

**Keywords:** audio deepfake, spoofed audio, spoof detection, deepfake detection, deepfake generation, misinformation, cybersecurity, artificial intelligence

## Abstract

A deepfake is content or material that is synthetically generated or manipulated using artificial intelligence (AI) methods, to be passed off as real and can include audio, video, image, and text synthesis. The key difference between manual editing and deepfakes is that deepfakes are AI generated or AI manipulated and closely resemble authentic artifacts. In some cases, deepfakes can be fabricated using AI-generated content in its entirety. Deepfakes have started to have a major impact on society with more generation mechanisms emerging everyday. This article makes a contribution in understanding the landscape of deepfakes, and their detection and generation methods. We evaluate various categories of deepfakes especially in audio. The purpose of this survey is to provide readers with a deeper understanding of (1) different deepfake categories; (2) how they could be created and detected; (3) more specifically, how audio deepfakes are created and detected in more detail, which is the main focus of this paper. We found that generative adversarial networks (GANs), convolutional neural networks (CNNs), and deep neural networks (DNNs) are common ways of creating and detecting deepfakes. In our evaluation of over 150 methods, we found that the majority of the focus is on video deepfakes, and, in particular, the generation of video deepfakes. We found that for text deepfakes, there are more generation methods but very few robust methods for detection, including fake news detection, which has become a controversial area of research because of the potential heavy overlaps with human generation of fake content. Our study reveals a clear need to research audio deepfakes and particularly detection of audio deepfakes. This survey has been conducted with a different perspective, compared to existing survey papers that mostly focus on just video and image deepfakes. This survey mainly focuses on audio deepfakes that are overlooked in most of the existing surveys. This article's most important contribution is to critically analyze and provide a unique source of audio deepfake research, mostly ranging from 2016 to 2021. To the best of our knowledge, this is the first survey focusing on audio deepfakes generation and detection in English.

## 1. Introduction

Deepfakes are content or material that are Artificial Intelligence (AI) generated or manipulated to pass off as a real audio, video, image, or text artifact, which are in some cases entirely generated by AI. The main difference between manual editing and deepfakes is that deepfakes are AI generated and closely resemble real life artifacts. The impact of deepfakes can be seen in society in the form of sensationalized political, general, social media, and also in the entertainment industry. Deepfakes and other internet-based misinformation have become more prevalent and have started impacting society in various ways as seen in these examples: (1) A video of Nancy Pelosi that slowed down her speech at a news conference to make it look like she was drunk (Funke, 2020)^1^. (2) A doctored photo of Joe Biden taken in 2019 was made to show him hiding in his basement from the public in a campaign video (Kessler, 2020)^2^. (3) A scammer created a voice deepfake impersonating a German executive to send a transfer of 220,000 Euros to a Hungarian supplier (Stupp, 2019)^3^. (4) Entertainment applications such as Spangler (2020)^4^ and Murphy and Huang (2019)^5^ that use visual deepfake techniques to change facial features and face swapping, respectively. The instance with Nancy Pelosi Facebook did not take down the video but it was labeled as partly false. However, claims keep materializing of her being intoxicated even though she has stated she does not drink alcohol (Funke, 2020). This could be as a result of a multitude of material from public appearances for those to create material to spread misinformation about her being intoxicated. Even though these false claims have been debunked, people still shared them, propagating a negative image of her.

Deepfakes have been used in political campaigns and the trend might continue. Another example in the recent news was when Mr. Biden was singing a couple of lines to Despacito but the video was lip synced to use profanity against the police (Herbert, 2020)^6^. Twitter labeled it later with a “manipulated media” warning, but this was not the case when it was first released so the damage may have already been done. This video was shared twice in attempts to portray Biden wanting to defund the police and being in opposition to law enforcement, which Biden has said that he does not stand for. Twitter has also labeled it as manipulated media since then (Saul et al., 2020).

Deepfakes can also be used to commit fraud. A CEO of a-K energy based firm who thought he was on the phone with his German boss had asked him to send a transfer of 220,000 Euros to a Hungarian supplier (Stupp, 2019). The criminals had used AI-based software to impersonate him. The exact name of the software that they used is unknown. However, this is a big deal because they were still able to receive one transfer of the money. They figured out that it was not the CEO before the second transfer, but the damage was done by then. This could become more prevalent in the next couple of years if there are no countermeasures for this. These examples may clarify the importance of deepfakes, their ability to impact people's lives, and why the attention toward it has increased exponentially. The number of articles regarding deepfakes from 2015 to 2022 has increased significantly. A huge increase happened between 2018 and 2019 (from 60 to 309). On 24th July, it was linearly estimated that the number of papers related to deepfakes will increase to more than 730 until the end of 2020 (Nguyen et al., 2021). However, the reality is more surprising than the mentioned estimate since we found there are 1,323 papers related to or referring to deepfakes that were published until the end of 2020. These numbers are obtained from https://app.dimensions.ai while searching deepfake keywords in the texts of the papers.

There are four broad categories of deepfakes that are suggested in this paper to simplify the multitude of types of deepfakes into more organized groups:

- Audio deepfake is AI-generated or AI-edited speech to sound as real.
- Text deepfake is anything that is textual on the internet or media that is AI manipulated or AI generated to look real.
- Video deepfake includes videos that are edited, synthesized by AI, swapping a persons face or reenacting their body movements, and altering content of speech using AI.
- Image deepfake is image that is AI generated mostly by generative adversarial networks and can also be AI edited, synthesized, and face swapped.

Most of the other surveys present the techniques, advancements, and challenges focusing on mainly image and video deepfakes. The lack of focus on audio deepfakes in surveys is a strong motivation for this article to concentrate on audio deepfakes, where it is heading and how to weaken its harmful effects. Therefore, the aims of this article are as follows:

- Summarizing most recent trends in each of the deepfake categories and shortcomings of defenses against them.
- Serving as a guide to generation as well as detection of **audio deepfake** architectures.
- Offering the countermeasures and future research directions in the field of **audio deepfake**.

**Scope:** In this work, we will present all deepfake categories obtained from more than 150 methods that we have surveyed. Since there are other surveys focusing on video and image deepfakes as well as text deepfakes, we pay more detailed attention to audio deepfakes' concepts and frameworks. We also provide a quick guide (Supplementary Table 1 in the Appendix) that can be used by those who are interested in audio deepfakes. We will not be discussing the details of the frameworks used for all of the deepfake categories. However, the most recent trends and frameworks are collected for each deepfake category (audio, text, video, and image). The rest of the paper is organized as follows: preliminaries are presented in Section 2. In Section 3, we present a systematic review on the scientific papers for each category of deepfakes, their generation and detection techniques, and the most recent trends. More details and the network schematics are provided in this section. We also provide detailed information and guidance for fake audio detection. Section 4 outlines the most commonly used English language datasets for fake audio detection. Section 5 presents a brief overview of the intuitions behind some of the important audio generative networks. Section 6 includes discussion and future directions. Finally, our conclusions are presented in Section 7. A quick guide of audio deepfake frameworks is provided in Supplementary Table 1 of Appendix. The summary (Supplementary Table 2, 3 in Appendix) provides some of the significant papers that are surveyed in this work and is also presented in the Appendix.

## 2. Preliminaries

To deeply understand different categories of deepfakes, their attacks, and their detection methods, we need to know some of the concepts that are the basis of deepfake technology. These concepts include understanding different networks as well as some necessary foundational definitions. Therefore, we cover these fundamentals here.

### 2.1. Deep learning vs. machine learning and artificial neural networks

Machine learning (ML) is a branch of AI. ML could be defined as an automated learning approach that enables computers to learn without being explicitly programmed (Xin et al., 2018). Deep learning (DL) is a type of ML that empowers computers to be trained through experience and understand the world in terms of a hierarchy of concepts (Goodfellow et al., 2016). The formation of neural networks that could mimic the human brain for analytical learning was a strong encouragement for the creation of DL (LeCun et al., 2015). Artificial neural networks can simulate the human brain mechanism to interpret different types of input data, such as images, sounds, and text (LeCun et al., 2015).

ANNs are computational systems based on the way in which the human brain works. ANN's intention, like other machine learning algorithms' goal, is solving problems with learning from data. ANNs are capable means for modeling complicated behaviors and patterns (Pijanowski et al., 2002; Grekousis, 2019). An ANN receives inputs and brings them to a network of nodes arranged in layers with connections and weights. The way the nodes are layered and connected is commonly called ANN architecture. In an ANN architecture, there is an input layer, an output layer, and one or more layers between them called hidden layers. Each layer contains neurons (nodes), and these neurons are linked through connections. A weight is assigned to each connection. The iterative process updates the weights to minimize the error and/or the occurrence of the stopping criteria (Grekousis, 2019). We can consider two major categories for ANNs: shallow ANNs and deep ANNs (Deng, 2014). Shallow ANNs refer to ANNs with one or two hidden processing layers, but deep ANNs have more than two hidden layers, so deep ANNs can model more complex problems (Deng, 2014; Grekousis, 2019). However, deep learning is not just regarding the number of hidden layers, it is also about the “entire architecture, processing functions, and regularization techniques that literally and dramatically change the ANN scenery” (Grekousis, 2019). For example, these problems could be solved using deep learning methods such as image classification, face analysis, and audio analysis (Goodfellow et al., 2016).

### 2.2. Networks used in deepfake generation and detection

Commonly, deepfakes are generated using combinations of four typical networks: encoder–decoder networks (ED), convolutional neural networks (CNN), generative adversarial networks (GAN), and recurrent neural networks (RNN). Brief explanations of each of the aforementioned networks are provided below.

**Encoder–decoder networks:** An ED contains two networks, one of them is an encoder network, and the other is decoder. The ED tends to summarize observed concepts (input) when it is trained like De(En(x)) since it has narrower layers toward its center (Mirsky and Lee, 2021). If the distribution of the x is X, the summary of x is En(x)= e that often is referred to as an embedding or encoding, and En(X) = E is referred to as the latent space (Mirsky and Lee, 2021). In deepfake technology, one may use multiple encoders or decoders and encoding manipulation to achieve a desired output (Maksutov et al., 2020).

**Convolutional neural networks:** The convolutional neural network was proposed for the first time by Lecun et al. (1998). Contrary to a fully connected (dense) network, pattern hierarchies are learned by a CNN that makes it able to work efficiently with image data. The hidden layers in a CNN could be multiple convolutional layers and the activation function followed by additional convolutions or ponds, fully connected layers, and normalization layers. All of them are called hidden layers because the activation function and final convolution cover their inputs and outputs functions (Song et al., 2021).

**Generative adversarial networks:** Generative adversarial networks were proposed for the first time in 2014 (Bengio et al., 2014). GAN is a system that includes two different types of neural networks, where the networks work in a zero-sum approach. There are a discrimination and a generation network. The generation network generates fake data, and the discrimination should estimate the probability of the fake data being real (de Rosa and Papa, 2021). Sometimes, the issue of a dataset shortage could be solved using GAN instead of CNN, and it has really helped in the early phase of COVID-19 pandemic to have a novel detection method (Loey et al., 2020). GAN has been used to generate new synthetic images of COVID-19, and developed to generate synthetic COVID-19 X-ray images (Rangarajan and Ramachandran, 2021). GAN includes some CNN layers with each forming the part of the discrimination and generative blocks (Bengio et al., 2014). Consider the discrimination network, the generation network, and a noise vector (the input), respectively, as D, G, and z. Then, D is trained to maximize the probability of classifying both training and generated data as real data. Simultaneously, G is trained to minimize log(1-D(G(z)) (de Rosa and Papa, 2021). Therefore, the two neural-networks systems compete in a zero-sum game, as shown below (de Rosa and Papa, 2021):


(1) $$min_{G}max_{D}C(D,G)=E_{x}[log(D(x))]+E_{z}[log(1-D(G(z))]$$

In Equation 1, C(D, G) is the loss function, D(x) is the probability of x to be considered as real in the discrimination network's classification, and note that x is truly real. *E*_*x*_ is the mathematical expectancy over all samples from the real data set X (de Rosa and Papa, 2021). G(z) is the generated fake data obtained from the z vector, so D(G(z)) is the estimated probability of the fake data to be real. *E*_*z*_ is the mathematical expectancy over all random generator inputs (de Rosa and Papa, 2021). However, there is a shortcoming of the equation above: in the early phase of the training, when the generative network has not generated enough and proper fake data, and the fake data is significantly different from the real data, the discrimination network refuses the fake samples with high probability. Thus, it gets trapped in local optimums. With training G to maximize D(G(z)), this local optimums' problem could be solved. GAN structure includes two different networks: Generator network and discriminator one. Some random input comes to the generator network which makes fake data. The fake data then goes to the discriminator network that also has some real data, and this network is supposed to classify the data as real or fake. The loss for generator and discriminator is also calculated.

**Recurrent neural networks:** Recurrent neural networks are a type of network that is trained with a sequence of training examples: (((*X*_1_), (*y*_1_)), ((*X*_2_), (*y*_2_)), …((*X*_*m*_), (*y*_*m*_))). The vectors *X*_*t*_ and *y*_*t*_ are representing input and output, respectively, *m* is the number of training examples, and *t* is the index of the training example. The steps below are performed in the RNN to calculate the output (De Mulder et al., 2015):


(2) $$\begin{array}{l} a_{j}\left(t\right)=\Sigma_{i=1}^{n_{I}}\alpha_{ji}x_{i}\left(t\right)+\Sigma_{i=1}^{n_{H}}\rho_{ji}h_{i}\left(t-1\right),\;j=1,\ldots ,n_{H} \end{array}$$

(3) $$\begin{array}{l} h_{j}\left(t\right)=F\left(a_{j}\left(t\right)\right),\;j=1,\ldots ,n_{H} \end{array}$$

(4) $$\begin{array}{l} b_{j}\left(t\right)=\Sigma_{i=1}^{n_{H}}\beta_{ji}h_{i}\left(t\right),\;j=1,\ldots ,n_{J} \end{array}$$

(5) $$\begin{array}{l} o_{j}\left(t\right)=G\left(b_{j}\left(t\right)\right),\;j=1,\ldots ,n_{J} \end{array}$$

The α_*ji*_, β_*ji*_, and ϕ_*ji*_ are weights (the parameters) of the network. As we know, like other ANNs, there are hidden neurons in the first hidden layer that receive the input vector *X*_*t*_ and calculate the linear combination of the individual components, in addition to performing a nonlinear transformation (F, activation function). The hidden neurons receive input values from both the input neurons and the hidden neurons. This is contrary to feedforward neural networks that only receive input values from the input neurons (De Mulder et al., 2015). The result will be sent to the output neurons that will calculate the output values *o*_*j*_(*t*). The variables *n*_*I*_ and *n*_*H*_ are the number of input layers and hidden layers, respectively. *F* and *G* are the nonlinear functions chosen by the user. The function F is often chosen as a Sigmoid function or as a hyperbolic tangent (De Mulder et al., 2015). For less-consuming computational time, the hyperbolic tangent function can be approximated by the hard tangent hyperbolic (Collobert et al., 2012). Recurrent Neural Networks have a great impact during the COVID-19 pandemic. For example, a paper used it to propose the state-of-the-art RNN models to predict the country-wise cumulative COVID-19 confirmed cases, recovered cases, and fatalities (ArunKumar et al., 2021). In contrast to some other types of ANNs, RNN is a powerful and robust type of artificial neural networks that uses existing time-series data for future data forecasting over a specified length of time (ArunKumar et al., 2021). Additionally, in some studies, one who works on sequential data like audio recognition may use another ANN such as CNN for feature engineering and extraction. Since RNN is an ideal model for solving sequential tasks (Sutskever et al., 2014), the output of the CNN phase, which is some feature vectors, is the input of a RNN, and the output of RNN could be classified data (Xie et al., 2021). In addition, RNNs without any other ANN networks are also used for many different problems, especially sequential ones. RNNs are widely used for speech emotion recognition by different researchers (Lee and Tashev, 2015; Tzinis and Potamianos, 2017; Li et al., 2021).

Table 1 shows the full forms of the acronyms used in this survey.

**Table 1:** The acronyms used in this paper.

**Acronym**	**Description**
AI	Artificial Intelligence
DL	Deep Learning
ML	Machine Learning
VC	Voice Conversion
SS	Speech Synthesis
TTS	Text-to-Speech
RNN	Recurrent Neural Network
CNN	Convolutional Neural Network
GAN	Generative Adversarial Network
ANN	Artificial Neural Network
DNN	Deep Neural Network
ED	Encoder-Decoder
Conv	Convolutions
ResNet	Residual Network
TCN	Temporal Convolutional Network

In the following sections, we categorize deepfakes. Then, we describe each of these types of deepfakes and ways by which they can be detected and created.

## 3. Deepfake categories

In this paper, deepfakes are categorized as audio, text, video, and image deepfakes. For each category, related papers are surveyed and the technology trends and frameworks are briefly discussed. As we mentioned earlier, audio deepfakes have been ignored in the surveys related to deepfakes. Therefore, to the best of our knowledge, this paper is the first survey focusing on generating and detecting audio deepfakes. In the audio deepfake section, we discuss some important frameworks in detail and provide readers with sufficient guidance for audio deepfake tools, some of which are shown in Supplementary Table 1 in the Appendix. In the following sections, we explain the deepfake categories.

### 3.1. Audio deepfakes

**Figure 1:**
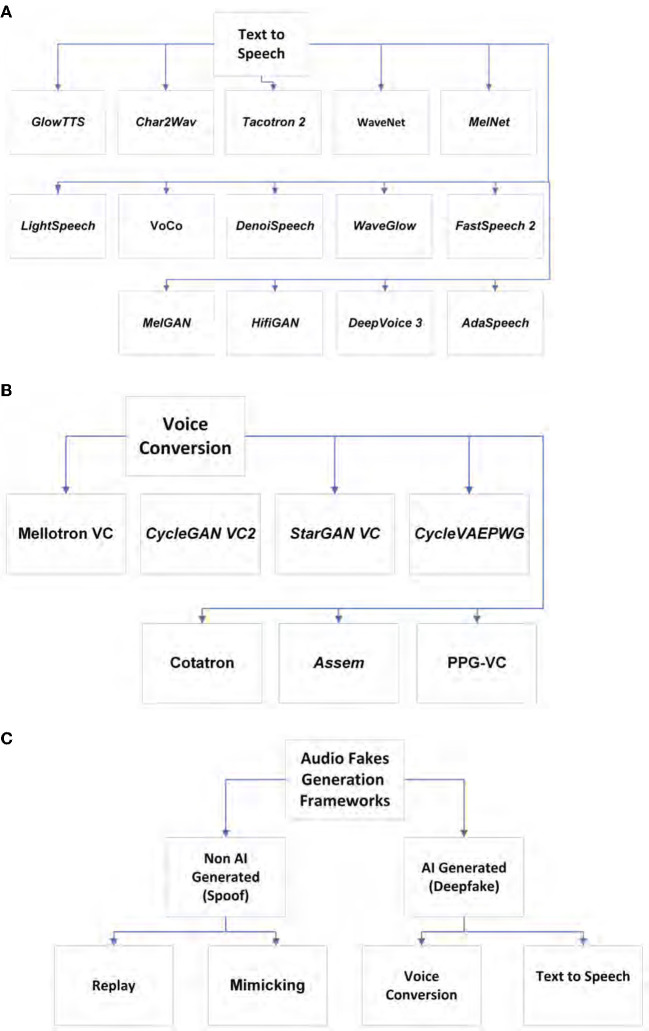
**(A)** Audio deepfake generation, text-to-speech. **(B)** Audio deepfake generation, voice conversion. **(C)** Audio fake generation frameworks.

**Figure 2:**
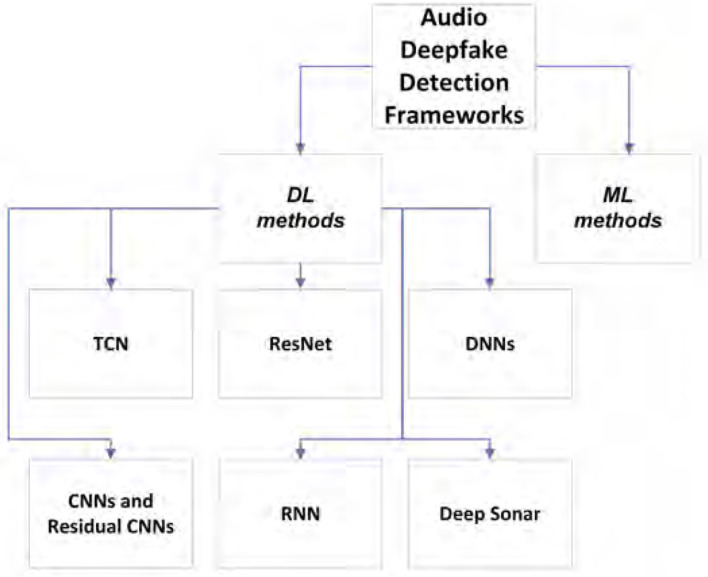
Audio deepfake detection frameworks.

Speech synthesis is the artificial speech that may be created by different technologies such as an audio deepfake. Audio deepfakes are AI generated or edited/synthesized to create fake audio that seems real. The subtypes of audio deepfakes are text-to-speech and voice conversion (including impersonation). As we will explain later, detecting audio deepfakes is really important since there have been some criminal activities using audio deepfakes in recent years. To achieve audio deepfake detection, one first needs to know the generation methods. Figure 1 shows audio deepfake **generation** methods, and Figure 2 shows audio **detection** tools and trends. Figure 1A indicates the frameworks that are often used in text-to-speech systems, and Figure 1B includes these frameworks for converting someone's voice. In addition, as we will discuss later, there are some non-AI-generated audio fakes, which we refer to as spoofs throughout the paper. There is another type of audio fakes called replay attack. This type of attack could be done simply using mobile phones or other available technologies. Although, one may use AI for a replay attack, it is not considered as a true deepfake due to the fact that one does not necessarily need AI to perform this type of attack. However, we also cover this type of audio in the literature because first, it is one of the most commonly used audio spoofing technique which has a lot of victims. Second, people use deep learning methods to detect this type of attack. Third, it may be also be created using AI-based technologies. Figure 1 shows the aforementioned categories. Audio fakes' methods are divided into two main categories: non-AI generated (replay attack) and AI generated or audio deepfake (text-to-speech and voice conversion). All of the aforementioned subcategories are discussed in detail in this section. The reader is also provided with the most recent and significant frameworks of each subcategory, as well as a quick guide for audio deepfake tools. In this section, both technical and theoretical information regarding different types of audio deepfakes and sufficient guidance for either generation or detection of audio deepfakes are provided. The summarized architectural schematics of some of the frameworks are given to help readers understand how they are designed. The purpose of these figures is to provide a quick and summarized look at different audio deepfake framework architectures. These frameworks' diagrams are color-coded, so orange and green refer to fake and real, respectively, also blue means using neural networks. After the architectural scheme, the most important methods related to each subcategory are given in the related sections. There are plenty of text-to-speech frameworks. A few of them are shown in the Figure 1A (Kim et al., 2020; Ren et al., 2020; Luo R. et al., 2021; Yan et al., 2021; Zhang C. et al., 2021).

#### 3.1.1. Non-AI generated: Replay attacks

Replay attacks are defined as replaying the recording of a target speaker's voice. The two subtypes are far field detection and cut and paste detection attacks (Pradhan et al., 2019). In far field detection replay attacks, the test segment is a far field microphone recording of the victim that has been replayed on a phone handset with a loudspeaker. Cut and paste detection system is if a recording is made by cut and paste short recordings to fake the sentence required by a text-dependent system (Pradhan et al., 2019).

**Attack:** Replay attacks are a threat to speaker verification systems because of low-cost recording devices and phones (Villalba and Lleida, 2011). Also, replay attacks can be used against voice assistants, which is dangerous especially since most voice assistants are used in the home (Pradhan et al., 2019).

**Figure 3:**
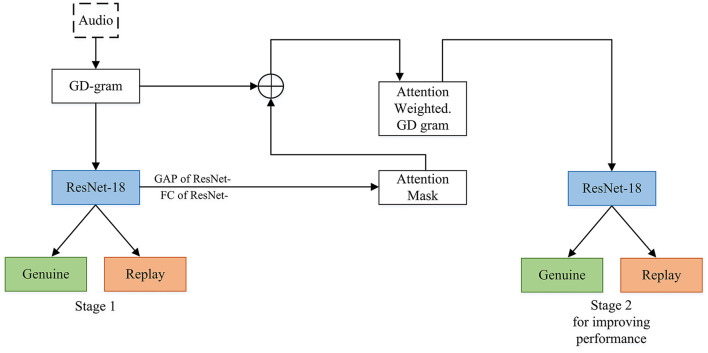
Replay attack end-to-end detection. The audio input comes to group delay grams which are novel time-frequency representations of an utterance. Also, the novel attention mechanism softly weights the GD grams. The ResNet-18 network and its GAP layer are used to provide attention maps for a second stage of discriminative training.

**Defense:** Some advantages of this category are that to defend against replay attacks one can use text dependent speaker verification (Villalba and Lleida, 2011). A current technique that detects end-to-end replay attacks is by using deep convolutional networks (Tom et al., 2018), which is shown in Figure 3. Figure 3 is an overview of the end-to-end replay attacks' detection framework by using deep convolutional networks (GD: Group Delay, GAP: Global Average Pooling, and FC: Fully Connected layer). Some of the replay attack detection systems have been proposed by working on the features which are fed into the network (Witkowski et al., 2017). Others have improved the networks used or have worked on both of the networks and features (Lavrentyeva et al., 2017; Nagarsheth et al., 2017; Gonzalez-Rodriguez et al., 2018; Huang and Pun, 2019, 2020; Lai et al., 2019; Li et al., 2019). Additionally, before the ASVspoof Challenge 2017 (Kinnunen et al., 2017; Lavrentyeva et al., 2017), there were only a couple of research papers done on replay attack, and after this challenge, more approaches for this attack were researched (Tom et al., 2018; Pradhan et al., 2019). Machine learning is not very effective for finding replay attacks because of overfitting due to the variability in speech signals (Li et al., 2017). It was found in the technique to detect replay attacks with deep convolutional networks that they were able to get a perfect Equal Error Rate(EER) of 0% for the development and evaluation set for ASVspoof2017 (Kinnunen et al., 2017). It means that the performance of the detection technique was really better than the previous ones; the best EER was 12% in the development set and 2.76% on the evaluation as stated in other literature (Tom et al., 2018).

#### 3.1.2. AI-generated audio fakes

Speech synthesis is one of the most important audio deepfake principles, and defined as artificially producing human speech by means of software or hardware system programs. One of the leading speech synthesis and audio deepfakes companies is Lyrebird-Descript, which uses deep learning models to generate 1,000 sentences in a second. It can also copy a voice fast, be adapted quickly to create what the creators want the phrase to be, and is language-agnostic. It can be used in the radio industry, traffic reports with auto voice overs, and streaming news bulletin systems, and the options are endless (Descript, n.d.). Unfortunately, SS systems can be used for nefarious purposes like creating a fake persona and stirring up political or societal drama. One needs a lot of processing power and data storage to create SS although the processing power is becoming less as the programs get better. It heavily depends on the speech corpus quality to make the system and, and it is expensive to create speech corpora (Kuligowska et al., 2018). It is probably easier to modify/update corpus than record a new one (Kuligowska et al., 2018). Sparsely spoken languages that do not have a standardized writing system make it hard to make a good speech synthesizer and linguistic components not easily available in all languages of the world also make it hard (Kuligowska et al., 2018). Another disadvantage is that SS systems do not recognize periods or special characters (Kuligowska et al., 2018). Ambiguities with homographs are the largest, which is when two words have different meanings but are written the same way (Kuligowska et al., 2018). Prosody, which means rhythm, stress, and intonation of speech, is one of the principles of a speech synthesizer system, and facilitates the implementation of complex psychological and phonetic impacts (Wolters et al., 2007). Prosody that changes the intelligibility and naturalness of speech synthesis systems is another disadvantage (Kuligowska et al., 2018). The problems can occur from prosodic bases, i.e., speech with little presence of emotions to the range of nuances aligned with an expression (Kuligowska et al., 2018). Accents can be hard to imitate because they lack dialect variation modeling (Kuligowska et al., 2018). Many synthesizers speak with a specific accent but a lot of it is not considered the “standard” accent of a certain language like how a person would sound (Kuligowska et al., 2018). One can sometimes tell it is not human-like because there is no breathing, laughter, pauses, and sighs among other things in human speech (Kuligowska et al., 2018).

##### 3.1.2.1. Speech synthesis (Text-to-speech)

Audio deepfake includes text-to-speech (TTS), which analyzes the text and makes the speech sound in line with text inputted using the rules of linguistic description of the text. An advantage of using text-to-speech is that it makes human-like speech from scratch and can be used for purposes like reading text and being a personal AI assistant, like Siri. Another benefit is that text-to-speech can offer different accents and voices instead of pre-recorded human voices. Text-to-speech takes text as an input; however, when a synthesis request is sent to TTS, a voice should be specified to speak the words; therefore, we can say that the SS-TTS models have been trained using audio samples of actual speeches. Besides the voice, some of the other aspects of the data output created by speech synthesis can be configured. TTS supports configuring the speaking rate, pitch, volume, and sample rate hertz (“Cloud Text-to-Speech basics^7^”).

**Figure 4:**
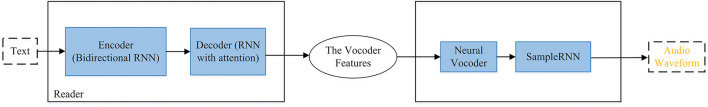
Char2Wav. It gets text as input, then brings it to an encoder and then decoder that both of them are RNN based, the output of this phase is linguistic features. The vocoder takes the linguistic features and gives audio.

**Figure 5:**
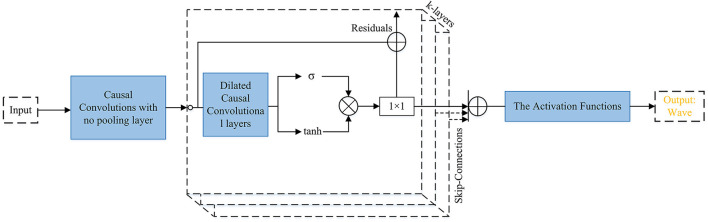
WaveNet. The input text is presented to causal convolutions, then the output comes to dilated convolutional layers, and then goes to gated activation units, and the activation functions. The Activation Function box includes two (Relu activation functions followed by 1 × 1 layers) plus a Softmax activation function. The figure above also shows the residual block used in WaveNet.

**Figure 6:**
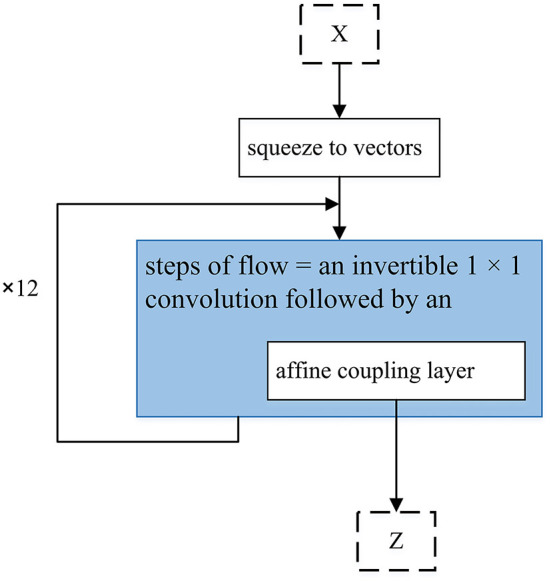
WaveGlow. The text input goes to a single network which is CNN based, and also tries to maximize the likelihood of the training data, and produces the audio output. *X* is a group of 8 audio samples squeezed as vectors.

**Figure 7:**
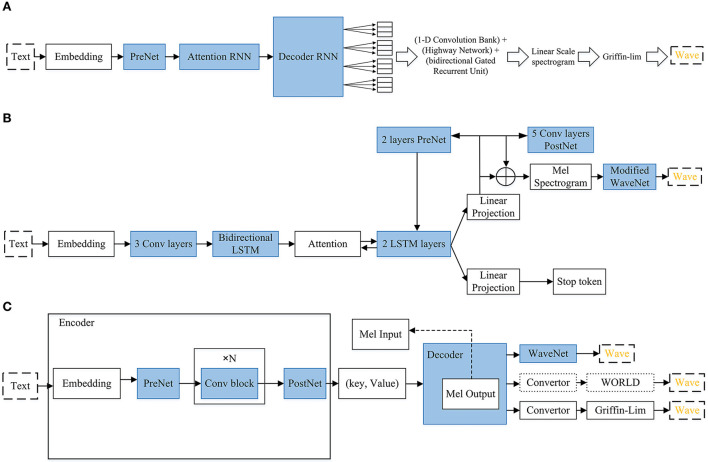
**(A)** Tacotron. The model takes input characters, then goes to some RNN based networks as well as a 1-D convolution bank + highway network + bidirectional GRU. Then, it gives the corresponding raw spectrogram as output, that is fed to the Griffin-Lim reconstruction algorithm for speech synthesizing. **(B)** Tacotron 2. Text characters are input then they go to embedding, PreNet, three convolutional layers, bidirectional LSTM, attention, 2 LSTM layers (recurrent sequence-to-sequence feature prediction network), Linear Projection, Modified WaveNet as vocoder. Then it outputs the wave. **(C)** Deep Voice3. Text characters are inputs, then they go for embedding, prenet, convolutional blocks and postnet that all of them are considered as the encoder. The output of this phase goes to the decoder which contains: prenet, attention blocks, causal convolutions, a fully-connected layer, and a binary final frame prediction. Then, it can use one of the existing vocoders for producing audio (WORLD, Griffin-lim and Wavenet).

**Figure 8:**
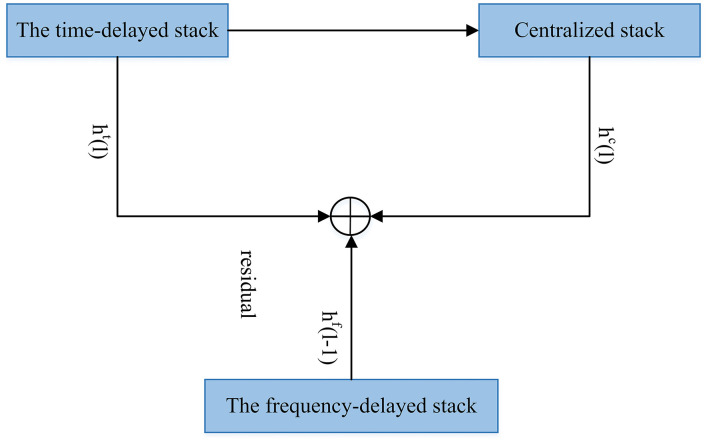
MelNet. The model gives the input to the three aforementioned stacks. The stacks extract features from different input sections to collectively summarize the entire context.

**Attack:** There are various generative networks that can be used to perform TTS attacks. Figures 4–8 are provided to help readers understand some of the architectures of speech synthesis TTS's generation frameworks and how networks are used. All of the architectural figures in this paper are the authors' interpretations of the proposed models based on the original architectures. Figure 4 shows Char2Wav that is an end-to-end speech synthesis generation framework. Figure 5 presents WaveNet (Oord et al., 2016), which is based on PixleCNN. The distribution of the conditional probability below is modeled by WaveNet:


(6) $$\begin{array}{l} p\left(x\right)=\overset{T}{\underset{t=1}{\prod }}p\left(x_{t}|x_{1},...,x_{t-1}\right) \end{array}$$

In this formula *x* = *x*_1_, …, *x*_*t*_ is a waveform, and *x*_*t*_ is an audio sample. The gated activation unit that is used in WaveNet is: tanh(*W*_f, K_*x)·σ(*W*_g, k_*x) where * and · respectively denote a convolutional operator and element-wise multiplication operator, σ(·) is a Sigmoid function, *k* is the index of the layer, *W* represents a learn-able convolution filter, *f* means filter and g denotes gate. In Figure 5, the text is input for the causal convolution with no pooling layer (the input and output have the same dimensions). Causal convolution is implemented by shifting the output of a normal convolution by a few timestamps. The dilated causal convolutions are used to increase the receptive field by orders of magnitude. The SoftMax activation function is used to model the conditional distribution p(x).

Figure 6 shows the overall structure of WaveGlow. Given that text-to-speech synthesis often includes two phases (encoder and decoder), WaveGlow focuses on the second phase. Therefore, WaveGlow is regarding transforming some time-aligned features, such as a mel-spectrogram obtained from encoder, into audio samples (Prenger et al., 2019). Input vectors (X) will be processed by the “steps of flow,” which includes an invertible 1 × 1 convolution followed by an affine coupling layer. The coupling layer is charged with the maintenance of the invertibility for the overall network. After network training, z values are randomly sampled from a Gaussian and run through the network. Tacotron 1 and 2 are presented in Figure 6. Tacotron, was originally suggested in 2017 (Wang et al., 2017). In Figure 6A, the system includes CBHG which is (1-D convolution bank + highway network + bidirectional GRU) (Lee et al., 2017). Tacotron is an end-to-end text-to-speech generative model that performs the entire synthesis of speech from the characters and the model can be trained from the ground up with random initialization given the text, audio pairs (Wang et al., 2017). Tacotron2 is an advancement of Tacotron and is a neural network architecture that achieves speech synthesis from text (Shen et al., 2018). It does this by means of a recurrent sequence–sequence feature prediction network, which maps character embeddings to mel-spectrograms which is followed by a modified WaveNet (Oord et al., 2016) model that acts like a vocoder to synthesize time domain waveforms for those spectrograms. Therefore, Tacotron 2 (Shen et al., 2018) system includes two components. The first component is a recurrent sequence-to-sequence feature prediction network with attention. The output of this component is a predicted sequence of mel spectrogram frames. The second component is a modified WaveNet vocoder. In Figure 6, attention means Location Sensitive Attention.

Figure 7 shows Deep Voice3 (Ping et al., 2018), which includes three parts:

- Encoder: residual convolutional layers are used to encode text into per-timestep key and value vectors.
- Decoder: (key, value) is used by decoder to predict the mel-scale log magnitude spectrograms. It contains causal convolutional blocks. Mel-band log-magnitude spectrogram is used as the compact low-dimensional audio frame representation. Two loss functions are used : L1 loss based on the output mel-spectrograms, and a binary cross-entropy loss based on the final-frame prediction. The following steps are performed in the decoder:

- Generating audio in auto-regressive manner.
- The decoder starts with Pre-Net, followed by a series of attention blocks and causal convolutions that generate queries to be utilized to attend over the encoder's hidden states.
- Finally, a fully connected layer gives the next group of audio frame to the convertor and a binary final frame prediction (will be the last frame of the utterance synthesized or not).
- Losses are calculated.

- Converter: A fully-convolutional post-processing network. Based on the chosen vocoder and the decoder hidden states, it predicts the vocoder's parameters. Dotted arrows mean the autoregressive process during inference.

The structure of MelNet (Vasquez and Lewis, 2019) is given in Figure 8. MelNet works in an autoregressive manner, and predicts a distribution element-by-element over the time and frequency dimensions of a spectrogram. The network includes different computational stacks that extract features from different pieces of the input. Then, these features will be collectively summarized to make the full context.

The stacks are described as follows:

- Time-delayed stack extracts features which aggregate Information from all previous frames. Multiple layers of multi-dimensional RNNs are used.
- Centralized stack contains an RNN. The RNN, at each time-step, takes an entire frame as input and gives a single vector that includes of the RNN hidden state as its output.
- Frequency-delayed stack uses all the previous elements in one frame. This is a one-dimensional RNN which moves forward along the frequency axis. This operates on a one-dimensional sequence (a single frame) and predicts the distribution of each element conditioned on all preceding elements and the outputs of the time-delayed stack.

The previous-layer's outputs of the frequency-delayed stack are *h*^*f*^(*l*−1), and *h*^*t*^(*l*) and *h*^*c*^(*l*) are current-layer's output of the time-delayed and centralized stacks, respectively. The outputs of the final layer of the frequency-delayed stack are used to compute the needed parameters for the audio generation.

Using neural network text-to-speech synthesis can make the speech audio in the voice of many speakers even those not in the training. This only needed 5 s (Jia et al., 2019). The first model to synthesize audio directly from text was Char2Wav which is end-to-end speech synthesis which has a reader and a neural vocoder to accomplish this (Sotelo et al., 2017). Baidu 3 Voice introduced a completely novel neural network architecture for speech synthesis and lets one use over 800 h of training data and synthesizes speech for over 2,400 voices, which is significantly more than other previously published text-to-speech models (Ping et al., 2018). Deep Voice 1 was the first to operate in real time for deep neural networks for text to speech, which is the foundation for end to end neural speech synthesis (Arık et al., 2017) and Deep Voice 2 (Gibiansky et al., 2017), was able to reproduce many voices using the same system. Moreover, most neural network based models for speech synthesis are auto regressive, meaning that they condition the audio samples on previous samples for long term modeling and are simple to train and implement (Prenger et al., 2019). A new company called Lyrebird-Descript AI uses deep learning to take bits of sound to transform speech and only needs a minute sample of someone's speech, like Barack Obama, to adapt to any voice (Descript, n.d.)^8^. Lyrebird, which is an AI research division within Descript, is faster than WaveNet for text-to-speech because it can generate 1,000 sentences in a second which is important for real time apps (Descript, n.d.)^9^. It can also copy a voice fast and is language agnostic. On the other hand, WaveNet listens to hours of raw audio to make sound waves sounding like a human voice. Voco is a speech synthesis framework, but includes text to speech and voice conversion of the text-based editing, pitch profile, and manual editing of length and amplitude (Jin et al., 2017). It was created by Adobe research and Princeton University students at the Adobe Max 2016 presentation and it sounds more human-like because of those features. It would have allowed those who are not pros to edit and search the transcript fast. Additionally, some widely published techniques for synthesis include Tacotron 2 (Shen et al., 2018), Tacotron (Wang et al., 2017), WaveGlow (Prenger et al., 2019), and MelNet (Vasquez and Lewis, 2019). Sometimes a combination of techniques have had papers written about them and one can find the GitHub repositories for this technique, which makes it more available and therefore people can help further the research (for example, Supplementary Table 1 in the Appendix, GitHub links). WaveGlow is a combination of Glow (Kingma and Dhariwal, 2018b) and WaveNet (Oord et al., 2016) that gives efficient, fast, and high-quality audio synthesis and does not need autoregression (Prenger et al., 2019). It also only needs a single network for implementation and can make high-quality speech from mel-spectrograms. MelNet is described as a generative model for audio in the frequency domain. It uses an expressive probabilistic model and multiscale generation procedure to create high-fidelity audio samples that snapshot the structure at timescales that time-domain models have not achieved (Vasquez and Lewis, 2019).

**Defense:** The text-to-speech detection systems are also used for voice conversion detection, so we review the detection methods for these two categories in the Voice Conversion Defense section.

##### 3.1.2.2. Voice conversion and impersonation

The last subcategory of audio deepfakes is voice conversion, which takes the speech signal by the first speaker, the source, and modifies it to sound like it was spoken by the second speaker, i.e., the target speaker. Voice conversion could be helpful for flexible control of speaker identity of synthetic speech in text-to-speech (TTS). A benefit of using voice conversion is it can help those with speech disorders, which is useful for rehabilitation medicine (Abe et al., 1990; Toda et al., 2016). For example, those with dysarthria can transform the vowels of the speaker into a vowel space of a speaker that does not have dysarthria, with the speech intelligibility greatly improved (Kain et al., 2007). It can also be used to personalize speaking and hearing aid devices and also speech-to speech translation. Voice conversion can be used for education by making a prosodically correct version of the utterances from foreign language learners to use in pronunciation training that is computer-assisted (Felps et al., 2009). It can be used for entertainment to take the emotion shown in a speech and transform it to another speech (Akanksh et al., 2016). It can take emotional speech and synthesize it from a typical reading that is neutral speech (Akanksh et al., 2016). The last example that can be used for entertainment is one can make multi-singer voices that vary with a conversion model used on a single-singer database utilizing direct waveform modification based on spectrum differential without vocoder based waveform generation (Kobayashi et al., 2014). Impersonation that can be considered as a kind of voice conversion is pretending to be another person for the purpose of fraud or entertainment. The advantages of impersonation include not having to pay voice actors for movies or TV shows and other uses for the entertainment industry. It can be used for readings of audio books with famous celebrity voices. Last, it is faster now to impersonate with new technology and one company called Overdub (Descript, n.d.) can do an impression of any voice with 1 min of sample audio. The possibilities are endless though and those are not the only instances where voice conversion can be used for good.

**Figure 9:**
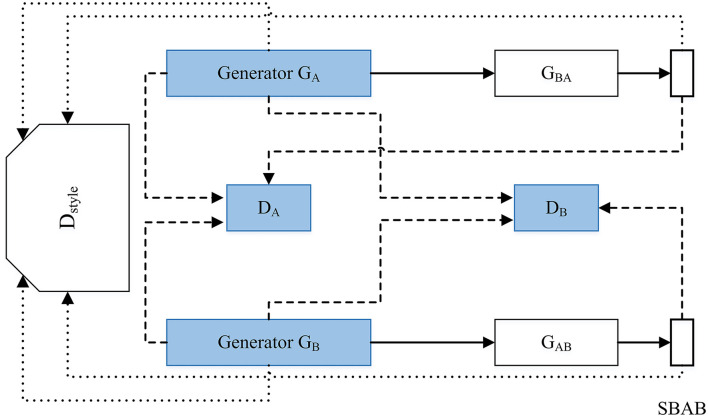
Impersonation using GAN. The GAN contains 6-layer CNN encoder and transposed6-layer CNN as its generative networks.The discriminative network contains 7-layer CNN with adaptive pooling.

**Attack:** Some drawbacks of voice conversion include phonetic issues, prosody, quality, similarity, overfitting, and threats to speaker verification systems so we need to push the anti-spoofing capabilities to improve in normal speaker verification systems (Wu and Li, 2014). Some disadvantages of impersonation using voice conversion are that there are many ways this can be used for fraud, such as the instance of impersonation of the German CEO. This was the first known instance of a deepfake voice scam and it could increase in the next couple years if this software becomes better and more available to the general public (Stupp, 2019). GANs can be used for voice impersonation like the framework that is presented in Figure 9 (Gao et al., 2018). This research (Gao et al., 2018) used a neural network framework to impersonate voices from different genders well with reconstructing time domain signals with the Griffin Lim method. This led to the model creating very convincing samples of impersonated speech. In Figure 9 *D*_*A*_ and *D*_*B*_ are the discriminators. The discriminator *D*_*style*_ determines if the original and transformed signals match the desired style. It uses the following style loss: *L*_*D style*_−*A* = *d*(*D*_*s*_(*x*_*A*_, *label*_*A*_)+*d*(*D*_*s*_(*x*_*AB*_), *label*_*B*_)+*d*(*D*_*s*_(*x*_*ABA*_), *label*_*A*_).

We note that voice conversion could be done without DL methods. One of the most important bases for voice conversion is the joint density Gaussian mixture model with maximum likelihood parameter trajectory generation considering global variance (Toda et al., 2007). This model is also the baseline of the open-source Festvox system that was the main voice conversion toolkit in “The voice conversion challenge 2016” (Toda et al., 2016). Voice conversion can be also based on other methods such as neural networks as well as speaker interpolation (Iwahashi and Sagisaka, 1995; Narendranath et al., 1995; Toda et al., 2007). When voice conversion is based on deep learning methods, it can be safely considered as a true deepfake. In recent years GANs are widely used for voice conversion due to their flexibility as well as high-quality results. For example, a singing voice conversion (SVC) framework using GAN is proposed (Sisman et al., 2019). They tried to convert a source singer's voice to sound like that of the target singer, without changing the lyrical content with the use of a GAN-based model (Sisman et al., 2019). In addition, since most of the VC algorithms are for using parallel data, Fang et al. (2018) has proposed a CycleGAN-based voice conversion system for nonparallel data-based voice conversion training. A CycleGAN is a GAN-based model for unpaired image-to-image translation, but Fang et al. (2018) used it to develop a voice conversion system that exceeded the performance of some state-of-the-art parallelVC methods. Also, StarGAN-VC is a framework that allows non-parallel many-to-many voice conversion by using a variant of a GAN (Kameoka et al., 2018). StarGAN also is originally an image-to-image translation system (Choi et al., 2018). Using text-to-speech networks in the structure of voice conversion may generate high-quality audio like ASSEM-VC (Kim et al., 2022), which is the state-of-the-art voice conversion system in terms of its naturalness. Due to the results of this study and their available code on Github, one can generate a very natural sounding voice ^10^. ASSEM-VC takes advantage of text-to-speech networks in its structure, the quality of the audio output depends on vocoder fine tuning in addition to the effect of pitch and linguistic features. In discussion with the authors it appears that despite the good output quality, the linguistic encoder of ASSEM system may not be robust to unseen speakers and utterances. The authors have solved this issue using Cotatron alignment (Park et al., 2020). Cotatron-VC is another voice conversion platform that estimates alignment between the input speech and its transcript in an autoregressive manner (Park et al., 2020). However, some noise in the input speech may corrupt the alignment estimated using Cotatron.

Supplementary Table 1 in the Appendix is provided, and it contains different audio deepfake tools, their summarized key features, and high-starred GitHub repository links. Therefore, one can use this quick guide table to generate their own audio deepfake samples, or to have the ability to detect them.

**Defense:** Since AI techniques are used in audio deepfake detection as we discussed in Section 2.1, we can categorize the detection methods into DL and ML groups (Almutairi and Elgibreen, 2022). Although it seems that the DL methods often outperform ML ones in terms of accuracy, it still worth trying both in terms of generalizability. Some ML models require more complex pre-processing phase, so in these cases DL models are a better choice. Khochare et al. (2022) used different Ml and DL methods on a new dataset called FOR (Reimao and Tzerpos, 2019). Machine learning models, such as Support Vector Machine, Random Forest, and K-Nearest Neighbors, could not achieve very high metrics, and the best of them stopped at 0.67 accuracy. There are a lot of different DL frameworks for audio spoof detection. ResNet, which was firstly used for image recognition, is utilized as the base of the audio spoofing (VC and SS) detection system (Chen et al., 2017). It is also improved to reduce EER metric as well as solve the generalization problem (Chen T. et al., 2020). Some also used temporal types of neural networks, namely Temporal Convolutional Networks (TCN) and achieved great results (Khochare et al., 2022). TCN has outperformed multi-layer perception in audio spoof detection (Tian et al., 2016). TCN has a good ability in capturing temporal dependencies in data (Chen Y. et al., 2020), which could be used in audio deepfake detection (Khochare et al., 2022). Some used RNN-based biLSTM networks to detect the deepfakes and have good performance (Arif et al., 2021). However, still TCN and ResNet seem better in terms of accuracy. In addition, some of the audio spoof detection systems have been extended by working on the features which are fed into the network (Balamurali et al., 2019). While others have worked on the networks used or both of the networks and features (Scardapane et al., 2017; Alzantot et al., 2019; Chintha et al., 2020; Rahul et al., 2020; Wang et al., 2020b; Luo A. et al., 2021). Therefore, besides the modeling phase, the features which are fed to the models are really challenging in the field of audio deepfake. Most of the features are the spectral features obtained from audio data, a comprehensive review of the different features which are used in audio deepfake detection could be found in Almutairi and Elgibreen (2022). Also, Blue et al. (2022) used a novel approach in terms of finding ideal feature set to be fed in different ML and DL models. They have constructed a mathematical model for simulating a speaker's vocal tract based on the amplitudes of specific frequencies present in their voice during a certain pair of adjacent phonemes (Blue et al., 2022). Although this study achieved a high accuracy, the drawback is they used fake samples generated based on Tacotron 2; therefore, it is needed to test the generalization of their method when using the other types of text-to-speech or VC networks.

**Figure 10:**
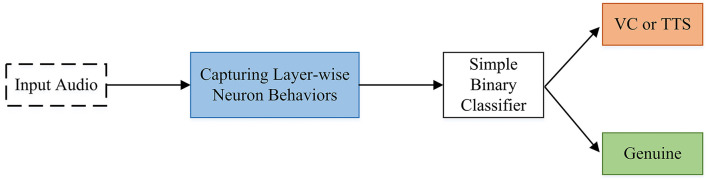
DeepSonar architecture for detecting AI-synthesized fake voices.

Some methods use the power of layer-wise neuron activation patterns with the assumption that they can capture the imperceptible differences between fake and real audios (Wang et al., 2020a). The study (Wang et al., 2020a) proposes an audio deepfake detection system called DeepSonar capable of both VC and text-to-speech samples. They achieved very good accuracy using a simple binary classification since their extracted features are quite distinguishing (Wang et al., 2020a). Figure 10 shows the overall architecture of DeepSonar.

### 3.2. Text deepfake

The text deepfake field is teeming with papers and techniques to create deepfakes; however, detection methods are catching up but not fast enough. One of the subcategories of a textual deepfake is **exposed fabrications**, which are those that are being fraudulently reported, like tabloids and yellow press that use sensationalism and eye-catching headlines to get more profit/traffic. Yellow press or yellow journalism, which has been popular and uses exaggerations and obvious falsification, can also be called fake news. A recent case example was from a Nebraska TV news fell for a scam call claiming that the post office was closed due to the coronavirus, spreading misinformation to the public and affecting those who need to use the post office (Smith, 2020). It is especially hard to know if something is an exposed fabrication on social media because people are not required to post where the information was sourced from and some people will just believe what the post says without checking other sources for themselves. For example, recently, the New York Times cited a gender blind tech study in February but published an editor's note later to say it could not confirm if the study was true and the recruiting firm Speak with a Geek who did the supposed study was not shown to have been published anywhere (Smith, 2019). Different misinformation tactics, especially AI-generated ones, will probably be more advanced and perhaps harder to detect in elections to come. A survey by Zignal Labs displayed that out of over 2,000 adults in the U.S., 86% do not always fact check articles read from a link on social media (Kanski, 2017). Also, 27% of respondents from the survey say they do not fact-check articles they share. Recently, Instagram has added a feature that shows that a content might be misinformation (Constine, 2019).

The next subcategory of textual deepfakes are **humorous fakes**. This type of a text deepfake is different from the others in its purpose, and intends to fool people using humorous fakes. There are different websites using this type of deepfake for providing entertainment (Yankovic, n.d.). The last subcategory of textual deepfakes is the **large hoax**, which is falsification or deliberate fabrication in mainstream media that attempts to deceive audiences that it is real news which can be picked up by traditional news outlets. For example, NBC San Diego, CBS Austin, and others published stories about tortillas having health benefits for certain types of cancer. The story was disproved by experts so it was retracted from the news sites (NBC News, 2019). If these hoaxes are not fact checked by mainstream news outlets or those spreading information on social media, it can be hard to know what information out there is fake. There are some tools coming out that will help journalists and front line workers fact check images, such as Google Assembler (Assembler, n.d.).

### 3.3. Video deepfake

Generally, video editing has been around since 1997 like in the movie Forrest Gump to digitally put in archival footage of JFK and manipulate his mouth movements (O'Sullivan, 2019). Later, deepfake technology made video editing more believable. Many YouTube channels like Ctr Shift Face (Ctrl-Shift-Face, 2021) post video deepfakes with increasing capabilities due to the new tools and the vast amount of training data that is available on the internet. There has been quite a few research papers on the creation and detection side of deepfakes. Also, the amount of deepfake videos has about doubled from a 2018 report from Deeptrace which said there were 7,964 videos on the web (Ajder et al., 2019). Their 2019 report counted 14,678 deepfake videos and the amount of deepfakes by the end of 2020 will probably have increased greatly (Ajder et al., 2019). Deepfake videos can also sow seeds of distrust in politicians or anyone for that matter. For example, in Malaysia a same sex video was released of the Minister of Economic Affairs Azmin Ali (Reuters Staff, 2019). The aide said the video was real but Azmin along with his supporters said it was fake and made to sabotage his career since having sex with the same gender is banned in Malaysia (Reuters Staff, 2019). The video was not proven to be fake by experts. This can make people disbelieve true facts because it is uncomfortable. This is called the liars dividend: the risk that liars will invoke deepfakes to escape accountability for their wrongdoing (Engler, 2019). The first subcategory of video deepfakes is **reenactment**, in which a person manipulates the identity to impersonate it and control what the identity says or does for the expression, body, dubbing, pose, and gaze. The expression refers to the reenactment which drives the expression of the face. An example of this is a video where the Mona-Lisa was smiling, talking, and moving in different positions (Zakharov et al., 2019). The mouth reenactment is also called ‘dubbing' or lip sync. The pose is when one head position is driven by another and the gaze reenactment is where the direction of the eyes and the position of the eyelids are driven by another. The last sub-categorization for reenactment is for the body which uses human pose synthesis or pose transfer which is like a facial reenactment (Mirsky and Lee, 2021). A benefit of reenactment deepfakes is if one can not dance, one can transpose a dancer's moves onto ones own prerecorded video to look like one can dance (Chan et al., 2019). It can be used for other modes of entertainment like the many lip sync videos, for example Jordan Peele making a video where he dubbed his voice over Barack Obama, since he can do good impressions. A group of researchers from the University of Washington in Seattle in 2017 also dubbed former president Barack Obama so his lips moved in time with words from a very different speech. They did this by training on many hours of his weekly address footage and using a recurrent neural network that would use the mapping from raw audio features to mouth shapes (Suwajanakorn et al., 2017).

**Video synthesis and editing** is when one creates a video without a target to base it off of. It can be beneficial as entertainment for those who see the video or made it. On the other hand, it can make people think that the video was not synthesized and is real. Editing and synthesis are very similar in the regards that you are creating a new video when editing while synthesis you are creating an entire new video. Editing is another subcategory in which attributes are added, removed, or altered, which can be regarding the person's facial hair, target clothes, age, weight, and ethnicity or it can be related to the background like adding a tree that was not there (Mirsky and Lee, 2021).

**FaceSwap:** The last category of video deepfakes is FaceSwap, which is when someone's face in an image or video is replaced with another persons face. Faceswapping can be used for entertainment and Disney has recently developed technology that makes the face swap quality even better so they could use it to have any actor/actress in their movies. For example, models from Deepfake Lab made images that were 256x256 pixels but Disney's resolution is substantially better with 1024 x 1024 pixels. To achieve this, they use progressive algorithm training, stabilization technology and lighting effects (Naruniec et al., 2020). Also, Ctrl Shift face (Ctrl-Shift-Face, 2021) and other YouTube accounts do many face swap videos with famous actors swapped in movies or other videos. For example, he used clips from American Psycho but used Tom Cruise instead of Christian Bale as the main actor. For another example, Mark Zuckerberg's face was swapped in a video made by an artist that wanted to draw attention to Facebook's privacy data scandals and to teach people about how easily digital propaganda can be made (Rea, 2019). It was very realistic looking and if the person did not know the context of the video or who it was made by, they would believe that it was real.

There also was an app made that has since been taken down called FaceApp that allowed anyone to recreate videos with their own datasets and had been widely used for Faceswap for non consensual porn. Even everyday people have fallen victim to deepfake porn because a young woman named Noelle Martin's was a victim of non-consensual porn. She became an advocate to prevent it from happening to others and there were no laws in place in New South Wales against this previously (Harris, 2019). She advocated for these protections and they were set in place in New South Wales in 2017 and in 2018 at the Commonwealth level and in Western Australia (Noelle Martin, 2019). There has been a plethora of research on face swapped images and videos and since the technology has been around and used since the early 2000s. Yet, the technology has gotten exponentially better and used more widely. The biggest trend of face swapped videos is deepfake porn which will continue to grow unless quick action for detection methods or prevention for this content allowed on Reddit is done. Also, a California Law AB 602 banned porngraphic deepfakes that were made without consent (Sierra, 2020). This allows state residents to sue anyone who uses a deepfake to place them in pornographic material without consent. Although there needs to be more laws in place like this in every state in the USA because it can happen to anyone in any state or country. It is very hard to trace the source since, in most instances, someone had made it in another country. Writing in new laws into policy can take a while and may not catch up to how fast deepfake technology is changing. Also these laws can protect people impersonating others that could ruin their reputation like pornographic deepfakes, which is 96% of deepfake videos according to research by DeeptraceLabs (Ajder et al., 2019). It is also important for companies like Youtube to have disinformation policies so people know what they are watching and seeing on social media that they can trust. Conversely, because of varying policies in other countries and with no legal jurisdiction, it can be hard to regulate deepfakes especially if it is created in another country. Snapchat had acquired the technology that helps make the filter with the facial mapping called Looksery by the same CEO of AI factory in 2015 (Spangler, 2020). An app called Zao (Murphy and Huang, 2019) has become very popular less skilled users can faceswap their bodies of movie stars and put themselves into well-known movies and TV clips. It was the most downloaded app among the Chinese apps over the weekend of 30 August. Zao (Murphy and Huang, 2019) is owned by Chinese hookup and live-streaming company Momo Inc. (Murphy and Huang, 2019). Earlier, the user agreement said that it had “free, irrevocable, permanent, transferable, and relicenseable” rights to the user made content (Murphy and Huang, 2019). Having rights to their face is a huge issue because the users did not know what it was being used for and possibly would license their face to other companies. Following that there was a great amount of negative reviews, with users complaining about the privacy issues (Murphy and Huang, 2019). WeChat also banned links to the app there being security risks (Murphy and Huang, 2019). Therefore, Zao (Murphy and Huang, 2019) has updated its terms and stated it will not use mini videos or headshots by users for reasons other than to improve the app or things pre-agreed upon the users. The deleted content by users will also be erased from the servers. A result of this app is that one can see how easily it is to mass distribute a deepfake app which raises concerns not just about the persons privacy but also ethical issues. The smooth integration into videos and memes make it stand out from other apps because of the series you can take with the photos where you blink and open your mouth to make a more realistic deepfake.

### 3.4. Image deepfakes

The last category discussed for deepfake technology is image deepfakes. **Faceswap:** One of the subcategories of image deepfakes is also faceswap. Faceswapping can also be difficult to identify it as a deepfake when the pictures artifacts are hidden behind the compression artifacts (Mirsky and Lee, 2021). Snapchat was the beginning of the face swap technology available to the public and there have been more platforms and the technology has gotten better ever since. Snapchat bought a deepfake AI startup that allows users to insert a selfie of themselves into a scene called Cameos that then sends the short looping video to friends (Spangler, 2020). Fakeapp is still the most popular face swapping app at the moment when it went viral around the world for showing people what they would look like when they were older and doing gender swaps (Murphy and Huang, 2019). Like other faceswapping apps, such as Zao (Murphy and Huang, 2019), there have been some privacy implications. **Synthesis and editing:** Another subcategory of image deepfakes is synthesis. Image synthesis can allow someone to make a new AI-generated image for personal reasons or for entertainment. Also another advantage is that neural textures can allow one to resynthesize new views of static objects and then edit the scene along with re-rendering dynamic animated surfaces. It is easy to make deepfakes GANs now more than ever and there have been instances of synthesizing images that use GANs. NVIDIA's 112 can make endless variations of the same image it generated. The results are very realistic and it uses data-driven unconditional generative image modeling (Karras et al., 2020). This opens an opportunity for artists because of all the different image variations can bring their ideas to the forefront. StyleGAN2 is also helping detect image deepfakes as it can see if the picture is generated by a network or not (Karras et al., 2020). In image synthesizing, new techniques allow one to combine the discriminative power of a deep neural network with classical MRF(markov random field) models based on texture synthesis which creates a more realistic looking image (Li and Wand, 2016). Also, GANs and auto-regressive networks can get good results when synthesizing individual images. Conditional GANs are the standard to do conditional image synthesis, and it can connect two different spheres like photorealistic imagery and lacking computer vision reconstructions (Thies et al., 2019). Editing images using photoshop tools have been used for many years. However, image editing using AI tools has proposed robust way to edit the images vastly. FaceApp is a newer mobile application that allows one to alter the age, smile, and change genders and claims to be the most advanced neural portrait editing tech available on the market. Since 2017, it has been downloaded by more than 100 million people around the world because of its interesting features. FaceApp also uses AI to age the photos, which helps with the quality of the photo. It also gained access to all of your photos, Siri, and search history which brings up questionable concerns about how much an app needs to access to work. When accepting FaceApp's terms of service you allow them “nonexclusive, royalty-free, worldwide, fully-paid, transferable sublicensable license to use, reproduce, modify, adapt, publish, translate, create derivative works from, distribute, publicly perform and display your User Content and any name, username or likeness provided in connection with your User Content in all media formats and channels" now known or later developed, without compensation to you (FaceApp, n.d.). The survey performed by Tolosana et al. (2020) has evaluated different image (focusing on face) manipulation as well as detection techniques.

## 4. Audio deepfake datasets

Dataset has a significant impact on the performance as well as generalizability of an audio deepfake detection system. For example, Blue et al. (2022) has separated the speakers in their training and test sets. This means that their model is evaluated by speakers whose voices have not previously been fed into the training model. Due to the importance of choosing a proper dataset, in this section, we briefly introduce the popular datasets in English that are highly used in audio deepfake detection:

**1. ASVspoof datasets:**

- **ASVspoof 2015:** This dataset contains text-to-speech and VC samples (Wu et al., 2015). Genuine speech is collected from 106 speakers (45 male, 61 female) with no significant channel or background noise. The fake versions of the real clips are generated using a number of different spoofing algorithms. The full dataset is partitioned into three subsets: the first for training, the second for development, and the third for evaluation (Wu et al., 2015). Since the newer versions of ASVspoof challenge datasets are available, one may not find enough benefits in using this dataset in new experiments.
- **ASVspoof 2017:** The primary technical goal of ASVspoof 2017 (Wu et al., 2017) was advancing research toward general spoofing countermeasures, especially for replay attack detection. The ASVspoof 2017 dataset contains a large volume of speech data collected from 179 replay sessions in 61 unique replay configurations. The number of speakers is 42 (Wu et al., 2017). A drawback of this dataset is that most of the speakers are not native English speakers. Although having diversity in the speakers is an advantage for a dataset, not having enough native English speakers may be somewhat limiting factor in terms of generalizability of the approach being evaluated.
- **ASVspoof 2019:** The ASVspoof 2019 (Wang X. et al., 2020) edition is the first audio spoof detection challenge that considered all three spoofing attack types (replay, text-to-speech and VC). They have separated the existing scenarios as follows: Spoofing attacks within a logical access (LA) scenario generated with the latest TTS-SS and VC technologies. Replay spoofing attacks within a physical access (PA) scenario. This dataset is useful in terms of peforming different types of analysis based on the different types of attacks. Third dataset includes 107 speakers (46 male, 61 female).
- **ASVspoof 2021:** This dataset (Delgado et al., 2021) includes the LA and PA scenarios (Wang X. et al., 2020) and an additional scenario called speech deepfake database. This scenario is similar to the LA task, but there is no speaker verification.

**2. Fake or real dataset**

FoR dataset (Reimao and Tzerpos, 2019) is a new dataset which contains multiple versions: version one is original synthesized files. Version two contains the same files, but balanced in terms of gender and class and normalized in terms of sample rate, volume and number of channels (Reimao and Tzerpos, 2019). Version three includes the version 2 files that are shortened in 2 s chunks. The last version is a re-recorded version of the third one. This type of rerecording can allow for testing scenarios where speech is received over a phone call or voice message (Reimao and Tzerpos, 2019). FoR dataset uses some high quality text-to-speech algorithms such as deep voice 3 (Ping et al., 2018) and Google wavenet (Oord et al., 2016). However, in this dataset no VC algorithm is used.

**3. WaveFake dataset:**

The dataset (Frank and Schönherr, 2021) consists of 117,985 generated audio clips (196 h total time). However, this dataset includes both English and Japanese samples. This dataset also does not include any VC algorithms. One advantage of this dataset is that they have used various state-of-the-art text-to-speech algorithms.

## 5. Intuitions behind the AI-generated audio

Some text-to-speech networks play the role of neural-based vocoder in voice conversion and other text-to-speech networks. Vocoder is a part of AI-generative networks which synthesizes waveforms based on acoustic or linguistic features obtained from previous steps in the network (Tan et al., 2021). Due to the high significance of vocoders in AI audio generative networks, in this section, we briefly explain some of the intuitive logic behind them.

Wavenet is one of the popular text-to-speech models also used as vocoder in other models. Wavenet is based on convolutional neural network's structure. WaveNet (Oord et al., 2016) models the distribution of the conditional probability. Given the waveforms, modeling the probability distribution helps to generate realistic audio samples.

The other important network is Wave-Glow (Prenger et al., 2019). WaveGlow is based on Glow (Kingma and Dhariwal, 2018a), and they are considered as normalizing flow which is a kind of generative model (Tan et al., 2021). WaveGlow trains model by minimizing the negative log-likelihood of the data, and calculates the likelihood directly.

Some text-to-speech networks, used also as vocoders, are GAN based networks, such as MelGAN and Hifi-GAN. To train the generative adversarial network (GAN), one may use different available formulas. For Example, MelGAN uses the least-squares (LSGAN) formulation (Mao et al., 2017).

## 6. Discussion and future directions

This section first presents the critical discussion, analysis, and summarization regarding the compiled works focusing on audio deepfake generation. Then, a summarization of the current techniques as well as future directions against deepfake is presented. Supplementary Table 2 in the Appendix summarizes the key papers related to audio deepfake surveyed. Supplementary Table 3 in the Appendix summarizes the key papers of the other types of deepfakes surveyed.

### 6.1. Deepfake generation

In deepfake generation, the most significant aspect is how believable it is to the victim, that means “deepfake quality.” The higher the quality, the more threatening and effective the deepfake is. In the following paragraphs, we discuss about the trade-off between quality and some of the other aspects regarding our main focus, **audio deepfakes**.

**Data vs. Quality (MOS):** The Mean Opinion Score (MOS) is “the arithmetical mean of individual ratings given by different users” (Santos, 2019). MOS has been used in many researches surveyed here to identify the quality of the audio. Given our evaluation of different audio deepfake frameworks' performance, the Mean Opinion Score (MOS) of the generated audio is better when the framework is trained using single speaker datasets (Oord et al., 2016; Ping et al., 2018; Kumar et al., 2019; Kong et al., 2020). It means that training the models using multi-speaker datasets to generate natural audio samples could be challenging. Some researchers may sample from a multiple-speaker dataset; for example Kumar et al. (2019) and Kong et al. (2020) first time have sampled six and second time nine different speakers from VCTK (Yamagishi et al., 2019) dataset, respectively. Also, for single speaker training, many frameworks use almost 24 h of audio in their dataset. MelNet (Vasquez and Lewis, 2019), which has used a 140 h single speaker dataset, as well as VoxCeleb2 (Chung et al., 2018) multi-speaker dataset, has a better performance than the previous works. The VoxCeleb2 dataset contains over 2,000 h of audio data with real world noise such as background music, laughing and cross-talk. In addition, the dataset is captured from speakers of 145 different nationalities including different accents, ages, ethnicities, and even languages. The researchers are highly recommended to used different multi-speaker data such as VoxCeleb2 dataset and evaluate the obtained generalization.

**Sampling Frequency (kHz) vs. Quality (MOS):** When the sampling frequency (sampling rate) of the audio deepfakes is less than 16 kHz, perceived speech quality of audio deepfakes drops significantly, and the higher sampling rate may give way to higher audio quality (Prenger et al., 2019). For instance, although the LibriSpeech (Panayotov et al., 2015) dataset contains a lot more data than VCTK (Yamagishi et al., 2019), Deep Voice 3 has significantly better quality on the VCTK dataset. One of the affecting factors could be the sampling rate which is 48 kHz for VCTK (Yamagishi et al., 2019), but just 16 kHZ for the LibriSpeech (Panayotov et al., 2015) dataset. For future research, the impact of different sampling rates on the audio deepfake quality could be investigated.

**Availability vs. Quality:** We also found that the more the availability and reproducibility, the more development the technology will have. The frameworks including their code as well as the datasets used that are available publicly (Sotelo et al., 2017; Wang et al., 2017; Ping et al., 2018; Shen et al., 2018; Vasquez and Lewis, 2019) are more likely to be used for nefarious purposes or research, so they will be more developed. However, we cannot suggest that all of the frameworks and their datasets be made publicly available since some people are always ready to take advantage of them for fraud. Some researchers with their published papers have chosen not to publish their detection methods so as to not help attackers know how they were able to detect the deepfakes. For example, eye blinking was a detection method for video deepfakes (Li et al., 2018) and once this was known the adversary made their deepfakes better so they blinked well. It is recommended that academic centers prepare a researching environment to share deepfake related frameworks and datasets with just researchersx.

Using other deepfake types for a certain type: as we could see, a framework that has been proposed for generation of a certain type of deepfake could be used for the generation of another type of deepfake with some changes. For example, CycleGAN and StarGAN are two frameworks for image deepfake generation that are used as the base of two audio deepfake frameworks (Fang et al., 2018; Kameoka et al., 2018), which can work with non-parallel data not just parallel ones. Data conditions for VC could be parallel or non-parallel. Parallel VC datasets refer to the datasets with utterances of the same linguistic content, but uttered by different people (Zhang J.-X. et al., 2020), but in practice, non-parallel VC which is more challenging is needed. It seems that more work should be done regarding audio deepfake frameworks using non-parallel data, and in this way, researchers may use image deepfake frameworks as the base of their proposed framework.

### 6.2. Future defense against audio fakes

All in all, researchers demonstrate that deepfake generation methods are more powerful and faster-developing than the prevention, mitigation and detection methods. The approaches that are mentioned in the following paragraphs offer a modest defense against deepfakes.

**Prevention:** For the prevention of deepfakes, some suggested blockchains and other distributed ledger technologies (DLTs) can be used for finding data provenance and tracing the information (Chauhan and Kumar, 2020; Fraga-Lamas and Fernandez-Carames, 2020; Ki Chan et al., 2020; Yazdinejad et al., 2020). Extracting and comparing affecting cues corresponding to perceived emotions from the digital content is also proposed as a way to combat deepfakes (Mittal et al., 2020). Some recommend the content be ranked by participants and AI regarding if it is fake or real (Chen et al., 2019). For future directions, deepfake prevention is the area that needs more attention. Especially, researchers could extend using DLTs for digital content traceability, as well as using effective computing to combat deepfakes.

**Mitigation:** If many of the detection tools are open source, it will make the generation tools better, which can be used for nefarious purposes. It is astute to have these tools open source so there is more research generated about these topics and collaboration but, on the flipside, it might be better to keep some detection tools proprietary only to people who need it like fact checkers for reporters. This is so those making the generation models, perhaps for nefarious purposes, would not know exactly what features make it easier to detect a deepfake like, for example, someone pointed out that deepfakes do not blink well (Li et al., 2018). Later this was fixed to blink, and this made the deepfake video quality better and harder to detect if it is a deepfake. Also, deepfake videos have better quality that it is hard to tell if it is real or not because of the matching of the speech, facial expression, movements, etc. especially with face-swapping. For audio deepfakes, it is not 100% likeness of a human but there have been improvements to make it sound like more natural speech instead of computer generated. Additionally, the journals as well as academic centers can make researchers who work on extending deepfake generation frameworks, propose a strong method for detecting the deepfakes generated by their frameworks (e.g., Chan et al., 2019 has proposed it for their framework “Everybody Dance Now”).

**Detection:** As we mentioned earlier, more work has been done regarding deepfake generation than detection. In the following sentences, we present a summary and future directions about the spoof detection systems focusing on **“audio deepfakes.”** In audio deepfake replay attack detection, some of the frameworks have been proposed by working on the features which are fed into the network (Witkowski et al., 2017). Others have improved the networks used or have worked on both networks and features simultaneously (Lavrentyeva et al., 2017; Huang and Pun, 2019; Lai et al., 2019). Another category of audio deepfake detection systems aims to detect speech synthesis as well as voice conversion. Most of them use different DNNs such as ResNet (Chen et al., 2017; Chen T. et al., 2020) to detect audio spoofing. Additionally, some of the audio spoof detection methods have been extended by working on the features which are fed into the network (Balamurali et al., 2019). While others have changed the networks used or have improved both networks and features (Scardapane et al., 2017; Alzantot et al., 2019; Chintha et al., 2020; Rahul et al., 2020; Wang et al., 2020b; Luo A. et al., 2021). Given the fact that one of the most important deepfake detection challenges is “generalization,” researchers are highly recommended to work on generalization by changing or improving both of the networks and features as well as defining different loss functions (Chen T. et al., 2020; Zhang Y. et al., 2021). While many researchers work on useful classification DL networks, people are highly encouraged to find more distinguishing characteristics to be considered as the input variables. They can go beyond spectral audio signal features like MFCCs, and look at perceptual or linguistic characteristics which may be different in AI-synthesized and genuine audios. The study (Blue et al., 2022) is really insightful in terms of the input features since they used the speaker's vocal tract which is a novel approach. Another interesting idea regarding the input features is using layer-wise neuron activation patterns instead of raw MFCCs (Wang et al., 2020a). However, the aforementioned approaches needs special pre-processing to get the desired features. The researchers are recommended to focus on how to get distinguishing characteristics with less complex pre-processing phase.

Given the categories, we summarize some of the most important references regarding audio deepfakes which are used in this survey in Supplementary Table 2 in the Appendix.

The references which are about the other deepfake types are summarized in Supplementary Table 2 in the Appendix. For text deepfakes, a very rich summarization is available (Guo et al., 2020; de Rosa and Papa, 2021); therefore, we only mentioned three new works in the text deepfake area below.

Additionally, for visual deepfakes (image and video), there are some more surveys (Zhang T. et al., 2020; Mirsky and Lee, 2021; Nguyen et al., 2021).

## 7. Conclusion

People not just in this research field but everyday people need to be aware of deepfakes and the harm they can cause to minimize the adverse effects. Also, we need to question what we see and hear online since the content can be misleading. The categories of deepfakes were broken down into four categories: audio, video, photo, and textual. There were also subcategories discussed in each of the main four categories along with the advantages, disadvantages, and summary of the methods for each subcategory. In addition, in this research, we have focused on audio deepfake generation and detection. We have provided a deep overview of how the technologies which are used to create or detect audio deepfakes work, and also the details of their architectures. We hope this survey serves as a guide for people who are interested in understanding and preventing malicious deepfakes, and those who want to use deepfakes for well-meaning purposes. More research needs to be done in the field of audio and text deepfakes, especially audio since there is already a plethora of detection for different categories of textual deepfakes, specifically in the category of fake news.

## Author contributions

ZK created all of the tables and figures, wrote some parts of the manuscript: sections 2, 4–6. Also, 50% contribution to the sections 1, 3.1, and 7. GW drafted the article, wrote some parts of the manuscripts mostly sections 3.2–3.4. Also, 50% contribution to sections 1, 3.1 and 7. VJ supervised all of the parts of this work. All authors contributed to the article and approved the submitted version.

## Footnotes

^1^ https://www.tampabay.com/florida-politics/buzz/2020/08/04/politifact-nancy-pelosi-doesnt-drink-so-why-do-false-claims-about-her-being-drunk-keep-going-viral/

^2^ https://www.youtube.com/watch?v=9PUfxZQa7WQ

^3^ https://www.wsj.com/articles/fraudsters-use-ai-to-mimic-ceos-voice-in-unusual-cybercrime-case-11567157402

^4^ https://variety.com/2020/digital/news/snapchat-acquires-deepfakes-startup-ai-factory-cameos-1203456055/

^5^ https://www.bloomberg.com/news/articles/2019-09-02/china-s-red-hot-face-swapping-app-provokes-privacy-concern

^6^ https://www.oregonlive.com/nation/2020/09/trump-tweeted-a-deceptively-manipulated-video-of-biden-playing-an-nwa-song-at-a-rally-biden-actually-played-despacito.html

^7^ https://cloud.google.com/text-to-speech/docs/basics

^8^ https://www.descript.com/lyrebird

^9^ https://www.youtube.com/watch?v=YfU_sWHT8mo

^10^ https://github.com/mindslab-ai/assem-vc
