# Supplementary material for: Audio deepfakes: A survey
Source: Front Big Data. 2023 Jan 9;5:1001063. doi: 10.3389/fdata.2022.1001063 (PMC9869423; doi:10.3389/fdata.2022.1001063)
Supplement: Supplementary file 1 [file Table_1.pdf]

**Table 1.** Quick guide for Audio Deepfake tools

| Type | Name | Ref | Sample | Key Features |
|---|---|---|---|---|
| Replay/ Detection | Replay attack end-to-end detection | (Tom et al., 2018) | https://mohitjaindr. github.io/pdfs/c20-interspeech-2018.pdf | Contains a visual attention mechanism on time-frequency representations of speeches that uses group delay features and ResNet-18 architecture.The model works perfectly with an Equal Error Rate of 0 percent |
| Synthesis TTS/ Creation | Char2Wav | (Sotelo et al., 2017) | https://github.com/ gcunhase/PaperNotes/ blob/master/notes/. char2wav.md | Reader (frontend): Bidirectional RNN, transform the text into linguistic features” Neural vocoder (backend): Conditional SampleRNN: takes the linguistic features as input and creates the corresponding audio. |
| Synthesis TTS/ Creation | Tacotron2 | (Shen et al., 2018) | https://github.com/ NVIDIA/tacotron2 | End to end Text to Speech model that uses recurrent sequence-to-sequence feature prediction network (for Embedding characters to mel spectrograms) and a modified WaveNet. |
| Synthesis TTS/ Creation | VoCo | (Jin et al., 2017) | https:// www.youtube.com / watch?v=RB7upq8nz IU | Not open source. Includes text to speech and voice conversion of the text-based editing, pitch profile, manual editing of length and amplitude. |
| Synthesis TTS/ Creation | WaveGlow | (Prenger et al., 2019) | https://github.com /NVIDIA/waveglow | It combines insights from Glow and WaveNet to be able to provide fast, efficient and high-quality audio synthesis, without the need for auto-regression. It uses only a single network trained using only a single cost function: maximizing the likelihood of the training data, which makes the training procedure simple and stable |
| Synthesis TTS/ Creation | Tacotron | (Wang et al., 2017) | https://github.com /Kyubyong/tacotron | End to end text to speech model, creates audio directly from text. |
| Synthesis TTS/ Creation | MelNet | (Vasquez and Lewis, 2019) | https://github.com/ Deepest-Project/MelNet | It is introduced as a generative model for audio which can capture longer-range dependencies for the first time in the Text-to-Speech area. MelNet couples a fine-grained autoregressive model and a multiscale generation procedure to jointly capture local and global structure. |
| Synthesis TTS/ Creation | Deep Voice 3 | (Ping et al., 2018) | https://github.com/ Kyubyong/deepvoice3 | A fully convolutional attention based neural for Text-to-Speech that can create high-quality audio samples. |
| Synthesis TTS/ Creation | Wavenet | (Oord et al., 2016) | https://github.com /ibab/tensorflow-wavenet | It uses Causal Convolutional layers and Dilated Causal Convolutional layers to create high quality audio deepfake. |

| Type | Name | Ref | Sample | Key Features |
|---|---|---|---|---|
| Synthesis TTS/ Creation | GAN based Speech Synthesis | (Saito et al., 2018) | https://github.com /r9y9/gantts | Statistical parametric method for speech synthesis based on GANs |
| Synthesis TTS/ Creation | HiFi-GAN | (Kong et al., 2020) | https://github.com /jik876/hifi-gan | A GAN based speech synthesis framework which outperformed a lot of the previous works. |
| Synthesis TTS/ Creation | MelGAN | (Kumar et al., 2019) | https://github.com /seungwonpark/melgan | non-autoaggressive so fast, fully convolutional with significantly fewer parameters than the other frameworks. |
| Voice Conversion/ Impersonation/ Creation | a GAN based model | (Gao et al., 2018) | Not Found | Transfering style from one speaker to another. Obtained from huge modifications on the DiscoGAN |
| Voice Conversion/ Impersonation/ Creation | CycleGAN-VC | (Fang et al., 2018) | https://github.com/ jackaduma/CycleGAN-VC2 | A VC system based on CycleGAN. A nonparallel VC method that only learns one-to-one-mappings |
| Voice Conversion/ Creation | StarGAN-VC | (Kameoka et al., 2018) | https://github.com/ liusongxiang/ StarGAN-Voice-Conversion | It has developed StarGAN (Choi et al., 2018) to a VC system that allows non-parallel many-to-many VC. There is a generator that takes an acoustic feature sequence instead of a single-frame acoustic feature as an input and outputs an acoustic feature sequence of the same length. (same as Kaneko et al. (2017) papers. |
| Voice Conversion/ Impersonation/ Creation | SINGAN | (Sisman et al., 2019) | Not Found | GAN-based model for singing VC. |
| Voice Conversion/ Creation | ASSEM-VC | (Kim et al., 2022) | https://github.com/mindslab-ai/assem-vc | Assembling TTS vocoders and achieved very good voice quality |
| VC and TTS/ Detection | - | (Chen et al., 2020a) | Not found | Overcoming the generalization challenge by using: 1) large margin cosine loss function (LMCL) 2) online frequency masking augmentation that forces the neural network to learn more robust feature embeddings. |
| VC and TTS/ Detection | - | (Zhang et al., 2021b) | https://github.com/ yzyouzhang/ AIR-ASVspoof | An attempt to detect unknown synthetic voice spoofing attacks using one-class learning. It compacts the bonafide speech representation and injects an angular margin to separate the spoofing attacks in the embedding space. |
| VC, TTS and Replay attack/ Detection | - | (Chen et al., 2017) | Not found | Inspired by the success of ResNet in image recognition, they used it for automatic audio spoofing detection, and reduced the EER by 18 percent |
