# Supplementary material for: Audio deepfakes: A survey
Source: Front Big Data. 2023 Jan 9;5:1001063. doi: 10.3389/fdata.2022.1001063 (PMC9869423; doi:10.3389/fdata.2022.1001063)
Supplement: Supplementary file 2 [file Table_2.pdf]

Table 2. Summarization of the **Audio Deepfake** papers surveyed

Name	Ref	Architecture	Dataset	Objectives	Metrics	Results
Replay attack end-to-end detection	(Tom et al., 2018)	Group delay, ResNet-18 (it also includes Global Average Pooling, Fully Connected layers)	ASVspoof 2017 (Kinnunen et al., 2017)	An end-to-end deep learning framework for audio replay attack detection.	EER	Outperformed previous related works with Equal Error rate= 0 percent
Wavenet	(Oord et al., 2016)	Causal Convolutional layers, Dilated Causal Convolutional layers	To measure WaveNet Audio performance: For Multi Speaker Speech Generation: VCTK is used, and the dataset contained 44 hours for 109 speakers. For TTS: The same single-speaker speech databases from which Google's North American English and Mandarin Chinese TTS systems are built. For music audio modelling: the MagnaTagATune and the YouTube piano datasets.	High quality audio deepfake generation in three areas: Speaker Speech Generation, TTS and music audio modelling.	MOS scale for quality	Generation of raw speech signals with subjective naturalness never before reported in the TTS area. A new architecture based on dilated causal convolutions are developed. When conditioned on a speaker identity, a single model can generate different voices using WaveNet. The architecture of WaveNet gives great results when tested on a small speech recognition dataset. It also is so good in generating other audio modalities such as music.
VoCo	(Jin et al., 2017)	Forced alignment algorithm for corpus preparation TTS selection and preparation using MCD metric Building a voice convertor Synthesis and blending	CMU Arctic dataset	Editing audios using texts. for example to insert a new word for emphasis or replace a misspoken word.	Mel-Cepstral Distortion (MCD) Euclidean distance, MOS scale for quality	A system is presented that can synthesize a new word or short phrase, replace or insert it in the context of the existing speech

Name	Ref	Architecture	Dataset	Objectives	Metrics	Results
Tacotron	(Wang et al., 2017)	PreNet, Attention RNN, Decoder RNN, CBHG (1-D convolution bank + highway network + bidirectional GRU), Griffin-Lim reconstruction	an Internal North American English	An end-to-end generative TTS model that synthesizes speech directly from text (characters)	Visual Comparisons, MOS scale for quality	An end-to-end generative TTS model that achieved 3.82 subjective 5-scale score on US English.
Tacotron 2	(Shen et al., 2018)	PreNet, three convolutional layers, bidirectional LSTM, attention, 2 LSTM layers (recurrent sequence-to-sequence feature prediction network), Linear Projection, Modified WaveNet as vocode	an Internal US English Dataset	A NN architecture for speech synthesis directly from text.	MOS scale for quality	MOS = 4.53 comparable with MOS= 4.58 that is assigned to a professionally recorded speech.
Wave Glow	(Prenger et al., 2019)	It uses only a single network and a single cost function: maximizing the likelihood of the training data	Amazon Mechanical Turk	Presenting a ”flow-based network capable of generating high quality speech from mel-spectrograms”.	MOS scale for quality	It produces audio samples at a rate of more than 500 kHz on an NVIDIA V100 GPU. The MOS of it shows that its generated audio is as good as the best public WaveNet audio sample.
Char 2Wav	(Sotelo et al., 2017)	The Reader: (bidirectional RNN as the encoder, attention-based RNN as decoder) followed by the vocoder (neural vocoder and sampleRNN).	For being conditioned on English phonemes and texts is VCTK. For Spanish text is DIMEX-100	An end-to-end model for speech synthesis	No metric. Just some samples are given	Not contain any comprehensive quantitative analysis of the results, but shows some samples and their corresponding alignments to the texts.

Name	Ref	Architecture	Dataset	Objectives	Metrics	Results
Melnet	(Vasquez and Lewis, 2019)	Entirely recurrent architecture. It has three stacks, respectively: time delayed (Multiple layers of multidimensional RNNs), the optional centralized stack (an RNN), the frequency-delayed stack: (a one-dimensional RNN)	For unconditional generation: (Blizzard, MAESTRO, VoxCeleb2) For TTS: (TED-LUM 3 and Blizzard)	To have a generative network that can capture high-level structure that emerges on the scale of several seconds (subsampled spectrogram) in addition to details and high resolution (an iterative upsampling procedure)	Which model generates samples with longer-term structure answered by some human evaluators.	Instead of 1D time domain waveforms, it modelled 2D time-frequency representations such as spectrograms. It enabled fully end-to-end TTS that can generate audio with longer-term structure.
Deep Voice 3	(Ping et al., 2018)	Encoder: (PreNet, Convolutional Blocks, PostNet), Decoder: (in an autoregressive manner: PreNet, Attention Blocks and Causal Convolutional layers, Fully Connected Layers) then it followed by the convertor and chosen vocoder.	Single-speaker synthesis: an internal US English dataset. Multi-speaker synthesis: VCTK and LibriSpeech (Panayotov et al., 2015)	A new high-quality framework (A fully convolutional attention based neural) for TTS. The common error modes of attention-based speech synthesis networks and ways to mitigate them. Comparing some waveform synthesis models with each other.	Training iteration and achieving convergence time, MOS scale for quality	It is compared with Tacotron and is really faster in training iteration time and achieving convergence. The achieved range of MOS for single-speaker synthesis: (3.62-3.78) The achieved range of MOS for multiple-speaker synthesis for VCTK: (3.01-3.44). and for LibriSpeech (2.37-2.89)
HiFi-GAN	(Kong et al., 2020)	Generator (CNN) and two D Discriminators: multi-scale and multi-period. Two losses are added during the training. A Multi-Receptive Field Fusion module is added to the G	LJSpeech dataset (Ito and Johnson, 2017)	Proposing a framework for efficient and high fidelity speech synthesis based on GAN	MOS scale for quality	It outperformed WaveGlow in the end-to-end setting and some other frameworks, but the quality of the ground truth is still better.

Name	Ref	Architecture	Dataset	Objectives	Metrics	Results
MelGAN	(Kumar et al., 2019)	GAN-based: G: (Convolutional layer, Upsampling layers , Residual Stacks with dilated convolutional block) . D: (Each discriminator block contains Downsampling layer as well as some convolutional layers)	LJSpeech dataset (Ito and Johnson, 2017)	Using GAN for producing high quality coherent waveforms)	Number of parameters, Speed (in kHz), MOS	Its pytorch implementation ran more than 2 times faster in real time on a CPU and 100x faster than realtime on a GTX 1080 Ti GPU, with no hardware specific optimization trick. It is comparable in quality to state-of-the-art high capacity WaveNet-based models, but not better than them.
Cycle GAN-VC	(Fang et al., 2018)	Extracting two types of features of speech: The mel-cepstrum, (fundamental frequency) and aperiodicity bands. They are converted separately. GAN and linear conversion.	ALAGIN Japanese Speech Database	A nonparallel VC method	MOS scale for quality and similarity	Outperformed Merlin-based baseline (parallel VC) significantly. Slightly better than the state-of-the-art GAN-based parallel VC method
Start GAN-VC	(Kameoka et al., 2018)	Extracting two types of features of speech: The mel-cepstrum, (fundamental frequency) and aperiodicity bands. They are converted separately. GAN and linear conversion	ALAGIN Japanese Speech Database	A nonparallel VC method	MOS scale for quality and similarity	Outperformed Merlin-based baseline (parallel VC) significantly. Slightly better than the state-of-the-art GAN-based parallel VC method.
SIN GAN	(Sisman et al., 2019)	In the training phase WORLD vocoder is used. The run-time phase includes WORLD vocoder, GAN and another WORLD vocoder.	NUS Sung and Spoken LyricsCorpus (NUS-48E corpus) (Duan et al., 2013)	Using GAN for SVC (Singing Voice Conversion)	MOS, Preference score	Outperformed the DNN-based traditional SVC model.

Name	Ref	Architecture	Dataset	Objectives	Metrics	Results
GAN for Imperso-nation	(Gao et al., 2018)	GANs for style transfer, GANs for voice mimicry. The generative network: (6-layer CNN encoder and transposed 6-layer CNN). The discriminative network (7 layer CNN with adaptive pooling.)	TIDIGITS	Presenting GAN-based model for voice impersonation	The signal-to-noise (SNR) ratio test using the standard NIST STNR method and the WADA SNR method	The WADA test results are around 100 db. The STNR shows the generated data has good quality.
A spoof detection system (ResNet-based)	(Chen et al., 2020a)	The input of the ResNet is 60-dimensional linear filter banks (LFBs) that are extracted from raw audio. In the training phase FreqAugmentlayer and large margin cosine loss are used; same training utterances are fed into ResNet to extract spoof embeddings. They are utilized to train the backend genuine-vs-spoof classifier.	1) ASVspoof 2019 logical access (LA)dataset. 2) A noisy version of the ASVspoof 2019 dataset. 3) A copy of the dataset that is logically replayed through the telephony channel .	Proposing a spoof detection system that overcomes the generalization challenge	EER	Reduced ERR significantly (from 4.04 percent to 1.26 percent)
A spoof detection system (ResNet-based)	(Chen et al., 2017)	Features: Constant Q Cepstral Coefficients and Mel Frequency Cepstral Coefficients Classifiers: Gaussian Mixture Models, DNN and ResNet	ASVspoof2017 (Kinnunen et al., 2017) dataset	Using ResNet for automatic audio spoofing detection	EER	Outperformed the best single-model system by reducing EER 18 percent relatively.

Name	Ref	Architecture	Dataset	Objectives	Metrics	Results
One-Class Learning Towards Synthetic Voice Spoofing Detection	(Zhang et al., 2021b)	It has proposed the OC-Softmax loss function to solve the generalization problem	The development and evaluation sets of ASVspoof2019 Challenge logical access scenario	To detect unseen voice spoofing attacks using one-class learning, and solve the generalization problem of the previous detection methods	EER, countermeasure (CM) score	It has achieved an EER of 2.19 percent, and outperformed all existing single systems (i.e., those without model ensemble)
