# Supplementary material for: Audio deepfakes: A survey
Source: Front Big Data. 2023 Jan 9;5:1001063. doi: 10.3389/fdata.2022.1001063 (PMC9869423; doi:10.3389/fdata.2022.1001063)
Supplement: Supplementary file 3 [file Table_3.pdf]

Table 3. Summarization of the works surveyed regarding the other Deepfake types

Text Deepfake						
**Name**	**Ref**	**Architecture**	**Dataset**	**Objectives**	**Metrics**	**Results**
DGSAN	(Montahaei et al., 2021)	The framework contains iterations that each new generator is defined based on the last discriminator. For each iteration, while not converged, the framework samples from real data and generates fake data. It considers the generator found in the last step is called Qold and tries to generate Qnew from it. It also uses a discriminator between the real distribution and Qold, and tried to make Qnew optimal	COCO Image Captions, EMNLP2017 WMT News, Chinese Poems	Solving the problem of the existing GAN based methods with generating discrete data.	NLL, BLEU, Self-BLEU, MS-Jaccard	A new GAN-based framework to generate discrete data in which there is no need to pass the gradient to the generator. It has really better performance in the MS-Jaccard metric.

Name	Ref	Architecture	Dataset	Objectives	Metrics	Results
Text GAIL	(Wu et al., 2021)	A generative adversarial imitation learning framework for text generation is presented that utilizes huge pre-trained language models (pre-trained GPT-2 and RoBERTa) to provide a reliable guiding signal in the discriminators of the GANs. A contrastive discriminator, and proximal policy optimization (PPO) is applied to improve text generation.	COCO Image Captions, EMNLP2017 WMT News	Providing a reliable guiding signal in the discriminators of the GANs for text generation. Improving text generation performance using GANs.	NLL, BLEU, Self-BLEU, Perplexity	Unlike most of the previous GAN text generation frameworks, TextGAIL obtained better performance in terms of both quality and diversity than the MLE baseline
FGGAN	(Yang et al., 2020)	A GAN-based model, but it utilizes a feature guidance module for text features extraction from the discriminator network. These text features are converted into feature guidance vectors which are fed into the generator network to enhance guiding signal. Text semantic rules are also formulated.	COCO Image Captions, Synthetic Data, Chinese Poems	Solving the problem of weak feedback (guiding signal)from the discriminator network to improve GAN-based text generation performance	NLL, BLEU	Better than existing baselines in terms of sentence quality

Video Deepfake						
**Name**	**Ref**	**Architecture**	**Dataset**	**Objectives**	**Metrics**	**Results**
Every body Dance Now	(Chan et al., 2019)	TPose Encoding: pretrained Open-Pose + global pose normalization. Pose to vid Translation: the updated GANs (for temporal smoothing and Face GAN) Optimizing GANs with the objective	62 subject set of short 1920 × 1080 resolution dancing videos from YouTube	a simple method for "do as I do" motion transfer	SSIM.: Structural Similarity. LPIPS: Learned Perceptual Image Patch Similarity. Pose distance is evaluated between input and target.	High quality deepfake videos
-	(Zakharov et al., 2019)	A Meta learning: Embeddor, a Generative network and discriminative network (GAN)	VoxCeleb1 and Voxceleb2	Creating personalized talking head models using just a few images of a person or even one image.	SSIM, CSIM, USER	It presented a framework with so few-shot capability that is considered a meta-learning GAN model. It has been trained on a huge dataset of video. Then, it can learn one or few shot learning of neural talking head models of unseen people.
-	(Suwajanakorn et al., 2017)	RNNs are used. A RNN learns the mapping from raw audio features to mouth shapes. Given the mouth shape at each time-instant, mouth texture is synthesized, then it is composed with proper 3D pose matching after retiming.	For training 300 weekly addresses spanning 2009 to 2016 are downloaded. Each address lasts about 3 minutes on average, therefore totally 17 hours of video are used	Synthesizing video from audio in the region around the mouth	More natural	High quality video deepfake of President Barack Obama speaking with accurate lip sync

Name	Ref	Architecture	Dataset	Objectives	Metrics	Results
Deferred Neural Rendering	(Thies et al. 2019)	TThe rendering network is based on a U-Net (Isola et al., 2017). Encoder:(some convolutional layers each with instance normalization and a ReLU activation). Decoder:(mirrors the encoder). A TanH activation as inPix2Pix (Isola et al., 2017) is used for the final output layer.	Synthetic sequence including 1000 random training views and a smooth trajectory of 1000 different test views on the hemisphere. For facial reenactment: They used 650 training images for Macron, 2400 for Obama, and 2400 for Sequence 17.	A system that combines the traditional graphic pipeline with learnable components of ML to use of imperfect 3D content for producing photo-realistic (re-)rendering	MSE	Neural Rendering is introduced for photo-realistic image synthesis based on imperfect 3D contents at real-time rates. Neural Textures are presented for novel view synthesis in static scenes and for editing dynamic objects.It is faster than regular reenactment, asking only a few milliseconds input for high-resolution output.
-	(Naruniec et al., 2020)	An Unsupervised learning and progressive training of multi-subject face swapping: Normalization all available examples to 1024X1024 resolution. 1) Embedding images using a common encoder, mapping back them to the pixel space using a desired decoder (a multi-way decoder allows for generating different outputs). 2) Face alignment and landmark stability 3) Contrast-Preserving, Multi-Band Compositing	They created their own dataset not publicly available.	High resolution face-swapping pipeline at megapixel resolution.	No special metric, but the results are more realistic in comparison to the other state-of-the art face-swapping mode.	The first method capable of rendering photo-realistic and temporally coherent results at megapixel resolution. The importance of progressive training for high-resolution face-swapping is proved. Providing a landmark stabilization procedure that mitigates the temporal instabilities in the high-resolution domain. It has been compared with three open-source approaches that were considered as the state of the art in facial appearance transfer

Image Deepfake						
**Name**	**Ref**	**Architecture**	**Dataset**	**Objectives**	**Metrics**	**Results**
NVIDIA Style GAN2	(Karras et al., 2020)	The style of the blocks includes: Modulation followed by Convolutional layers (3X3), then normalization. Several changes are done on the original StyleGAN (Karras et al., 2019) to obtain the revised architecture. For example: the addition of biases and the noise broadcast operation are moved to the outside active area of a style. The revised architecture makes it possible to replace instance normalization with a "demodulation" operation.	FFHQ and LSUN CAR	Revising and improving the StyleGAN framework (Karras et al., 2019).	FID, PPL, Precision, Recall	The proposed framework (StyleGAN which was state-of-the-art in data-driven unconditional generative image modeling) is improved and analysed in terms of existing distribution quality metrics as well as perceived image quality.
-	(Li and Wand, 2016)	GAN: Generative network: based on Markov random field (MRF) models. Discriminative network: trained deep convolutional neural networks (dCNNs). GitHub Code: https://github.com /chuanli11/CNNMRF	-	Synthesizing 2D images	No special metric, but the results are better than previous works especially in art-work synthesis	The method can transfer both photorealistic and non-photorealistic styles to a new image. The combination of the discriminative power of a deep neural network with classical MRFs based models gives high-quality image synthesis.
